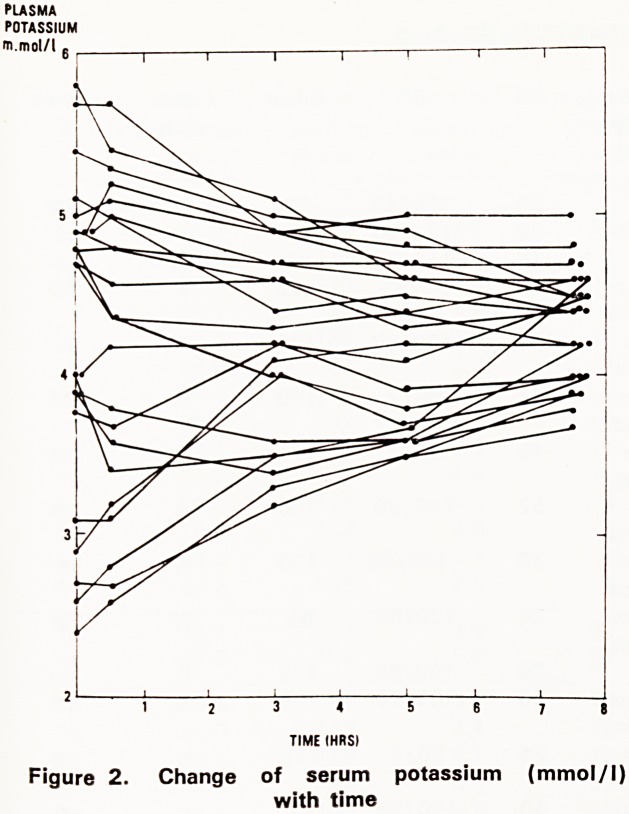# Low Dose Insulin Regimen for Diabetic Coma

**Published:** 1976

**Authors:** C. M. Asplin

**Affiliations:** Southmead Hospital, Bristol

## Abstract

Twenty-one patients with 22 episodes of diabetic "coma" have been treated with a fixed insulin, potassium and fluid regimen. Despite a wide range of clinical and biochemical features that may influence the response to such a treatment regimen such as age, acidosis or infection, the response as monitored clinically and biochemically was remarkably uniform.


*Bristol Medico-Chirurgical Journal. Vol. 91 No. 339/340*

### Experience in a District General Hospital using a Low Dose Insulin Regimen for Diabetic Coma

**Dr. C. M. Asplin, MB, ChB, MRCP**
Southmead Hospital, Bristol

Present address: Department of Medicine, Bristol Royal Infirmary, Bristol BS2 8HW

**Summary**

Twenty-one patients with 22 episodes of diabetic "coma" have been treated with a fixed insulin, potassium and fluid regimen. Despite a wide range of clinical and biochemical features that may influence the response to such a treatment regimen such as age, acidosis or infection, the response as monitored clinically and biochemically was remarkably uniform.

**Introduction**

Low dosage insulin regimens for diabetic "comas" have recently attracted considerable attention (Alberti, Hockaday and Turner, 1973; Page et al, 1974; Kidson et al, 1974; Semple, White and Manderson, 1974). These regimens have all originated from established diabetic centres, however the simplicity of these regimens, especially the intra muscular regimen (Alberti et al, 1973), would make them suitable for use in district general hospitals. In such hospitals there are often large medical "takes" and there may be problems in the provision of medical, nursing, intensive care or laboratory staff. Described below is the experience gained in a ten month period in a district general hospital of the application of a low dose insulin regimen in patients with diabetic coma or precoma who were admitted during the general medical intake.

**Patients and Methods**

Twenty-one patients, one patient with two separate episodes, were studied, all with a severe disturbance of their carbohydrate metabolism. There were ten men and eleven women with ages ranging from 17 to 84 years. Details of the patients are given in Tables 1 and 2. Fifteen patients were under 45, thirteen of whom were ketoacidotic, and six were over 65, none of whom were ketoacidotic. Plasma urea and electrolytes, blood glucose and arterial pH were determined by routine laboratory methods. A "Ketostix" reading of the plasma at 15 seconds was used as an assessment of ketoacidosis, 3+ samples indicating an acetoacetate concentration greater than 1.6 mmol/1 (Alberti and Hockaday, 1972). Plasma osmolarity (Osm) was calculated from the formula mOsm/l= 2 (Plasma sodium +potassium) +plasma urea+blood glucose (all measurements expressed in S.1 units.) Values of plasma osmolarity greater than 330 m Osm/l were judged to represent hyperosmolarity.

*Received for publication 1975*

**Clinical details (Table 1)**

On admission thirteen patients had a disturbed conscious level, five being in coma (no response to painful stimuli), three in pre-coma (purposeful response to painful stimuli) and five were drowsy but obeyed commands. The remaining nine were fully conscious. Five were hypotensive with systolic blood pressure under 90 mmHg. None were seriously hypothermic and seven had temperatures over 38°C. Four were newly diagnosed diabetics, all being in the younger age group. The remainder of this group were already established on either Lente insulin or a soluble and isophane mixture. In the elderly group only one was on insulin, three were on chlorpropamide and two on diet alone. The apparent precipitating factors of diabetic coma are shown in Table 1. Eight had proven infections, four of these of the chest and two of the urinary tract. Two patients had fractured femurs, one with a deep venous thrombosis and the other with a cerebrovascular accident. Another patient had sustained a myocardial infarction.

**Biochemical results (Table 2)**

The blood glucose on admission ranged from 11.1 to 81.0 mmol/l. Six patients had arterial pHs of less than 7.10 and ten had plasma bicarbonate levels of less than 10 mmol/l. Fourteen patients were ketoacidotic. The initial plasma sodium levels ranged from 128 to 158 mmol/l and the plasma potassium from 2.4 to 5.8. Plasma urea ranged from 6 to 25 mmol/l and thirteen were hyperosmolar.

**Treatment regimen**

**Fluids.** An immediate intravenous infusion was set up and 1 litre of saline (0.154 molar) given in 30 minutes, another litre over the next hour, followed by a litre over 1½ hours and then a litre two hourly for 4 hours. This gave a total of 5 litres of saline in 7

hours. For the elderly group 0.078 molar sodium chloride was used instead. After 7 hours the fluid was changed to 4.8% dextrose and 0.031 molar saline and approximately 3-4 litres per 24 hours was given until adequate oral intake was established.

**Potassium.** None was given in the first half hour but thereafter it was given in the intravenous infusion bottle as potassium chloride at the rate of 25 mmol per hour, over the first 7 hours. After this further supplements were given as 25 mmol per litre bottle and as soon as possible by mouth.

**Insulin.** 30 minutes after the beginning of the intravenous fluid therapy 10 units were given intra-muscularly, then every hour for 6 hours. Subsequently a "sliding scale" regimen of subcutaneous insulin was adopted based on 6 hourly urinary sugar estimations by a Elimitest.

**Heparin.** 5,000 units 12 hourly, subcutaneously, of heparin was used in five of the elderly patients and 40,000 units per 24 hours by intravenous infusion, in patient number twenty who had clinical evidence of a deep venous thrombosis.

**Bicarbonate.** This was used on only three patients, numbers six, seven and thirteen, and was given as a single 15 minutes intra-venous infusion of 100 mmol of sodium bicarbonate as a half-molar solution. The indications were very severe, distressing hyperventilation and abdominal pain which were thought to be as a result of the acidosis.

**Monitoring**

The progress of all patients was monitored clinically, with special regard to conscious level, respiratory rate and blood pressure. Blood glucose, plasma electrolytes and ketone bodies were measured four times during the first 7 hours at 30 minutes, 2 hours, 5 hours and 7 hours after admission. Where the arterial pH was markedly lowered this was serially measured as above until a return towards normality was detected.

**Results**

**Mortality**

Patient number twenty had a cardiac arrest 3 hours after admission, post mortem showed death due to a massive pulmonary embolus.

**Conscious level**

There was a rapid lightening of conscious levels and by 7 hours all were judged to have normal conscious levels.

**Blood pressure**

All the patients had systolic pressure greater than 110 mm Hg by 2 hours.

**Blood glucose** (Fig. 1)

The initial ½ hour infusion of 1 litre saline without insulin produced a fall in the blood glucose of between

**Figure 1. Change of blood glucose (mmol/l) with time**

0.5-7.5 mmol/l. By 7 hours all blood glucoses were in the range of 5.2-14.1 mmol/l except patient number twenty-two whose level was 2.7 mmol/l.

Rates of fall of blood glucose when insulin was started ranged from 0.5-16.0 mmol/l per hour. There seemed to be no correlation between the rate of fall of blood glucose and the height of initial blood sugar, the degree of ketoacidosis, the arterial pH, the clinical state, the presence of infection or the existence of previous insulin treatment.

**Ketostix readings**

By 2-4 hours all patients had Ketostix readings of less than 2+.

**Plasma bicarbonate**

By 7 hours all plasma bicarbonate levels were over 18 mmol/l and all arterial pHs where measured were greater than 7.30. When bicarbonate had been given the hyperventilation and abdominal pain were rapidly abolished.

**Plasma potassium** (Fig. 2)

The levels of plasma potassium returned gradually to normal whatever the starting value; no rapid fluctuations were noted and at 7 hours the range was 3.7 to 5.0 mmol/l.

**Figure 2. Change of serum potassium (mmol/l) with time**

**Plasma sodium**

The range of plasma sodium levels at 7 hours was 132-149 mmol/l.

**Plasma urea**

At 7 hours the levels of plasma urea ranged from 4.3-11.8 mmol/l.

**Discussion**

The rationale for the use of low dose insulin regimens has been fully discussed and compared with high dosage regimens (Alberti et al 1973). Special emphasis has been laid on the maintenance of the plasma potassium level and the less pronounced rise in blood lactate and growth hormone (Page et al, 1974 Alberti et al, 1973). In this series the low dose intramuscular insulin regimen was chosen because of its simplicity and suitability for use in a district general hospital. The patients studied covered a wide range of problems which might affect the success or failure of the regimen. Nearly one third of the patients were elderly, nearly one half had not received insulin before and over one half had some disturbance of conscious level. Five of the patients were hypotensive, a third had temperatures over 38°C, 8 had proved infections and one a myocardial infarction. There was a very wide initial range of biochemical findings such as blood glucose, acidosis, osmolarity and serum potassium levels. However, an identical regime was used for all patients except that those over 65 had fluid replacement over the first 7 hours with 0.073 molar instead of 0.154 molar saline.

The blood glucose response in a low dose insulin regimen is known to be independent of acidosis or previous insulin therapy (Alberti, Hockaday and Turner, 1973). This was confirmed in this series but in addition the presence of infection did not appear to influence the rate of fall of blood glucose. Although there was a marked variation in rapidity of fall of blood glucose, with fluid alone and after insulin treatment was started, by 7 hours all of the patients except one had blood glucose levels in the range of 5.2-14.1 mmol/l. suitable for commencement of a 'sliding scale'. In only one patient was a rather low blood glucose (2.7 mmol/l) encountered. Similarly, despite a wide range of initial plasma potassium levels, potassium replacement at a standard rate of 25 mmol per hour resulted in a satisfactory return of values to the normal range. There were rapid improvements in ketonaemia, bicarbonate and arterial pH levels. Intravenous bicarbonate was used only on three occasions to treat severe distressing acidotic symptoms (hypervenilation and abdominal pain). The same volume of intravenous fluid was given to young or old and hypo- or normotensives. Central venous pressure lines were not used but no patient developed clinical evidence of fluid overload.

Heparin was used where indicated clinically in full intravenous dosage, in the rest of the elderly group it was used in low dosage subcutaneously as a prophylactic measure against venous thrombosis.

In conclusion, the uniformity of clinical and biochemical improvement in 22 patients, with a wide variety of clinical and biochemical features of diabetic coma, during treatment with a low dose intramuscular regime makes the treatment suitable for hospitals with limited biochemistry facilities. This uniformity also means that requests for biochemical investigations can be kept to a minimum.

**Acknowledgements**

To Dr. J. Verrier Jones for permission to present patients under his care and to Dr. M. Hartog for advice and encouragement.

### TABLE 1

### ADMISSION DETAILS OF PATIENTS STUDIED

| Patient Ketoacidotic | Age | Sex | Duration DM (yrs.) | Previous Treatment | Precipitating factor | RR | BP | Pulse | Consc. state | Temp. °C |
|---|---|---|---|---|---|---|---|---|---|---|
| 1 | 17 | M | 3 | 36 MIX | 'Flu' | 36 | 100/50 | 88 | PC | $38^{5}$ |
| 2 | 29 | F | 7 | 48 MIX | Defaulter | 32 | 130/80 | 120 | D | $36^{5}$ |
| 3 | 42 | F | 18 | 28 L | UTI | 28 | 110/75 | 92 | D | $37^{5}$ |
| 4 | 37 | M | 6 | 68 L | Gastro-enteritis | 44 | 90/60 | 125 | C | $36^{5}$ |
| 5 | 32 | F | 12 | 92 MIX | Pneumonia | 36 | 100/50 | 120 | PC | $38^{5}$ |
| 6) | 34 | M | 8 | 66 L | None found | 44 | 60/? | 160 | N | 36 |
| ) 7) | | | | | None found | 40 | 80/? | 170 | N | 36 |
| 8 | 28 | M | — | — | Carbuncles | 32 | 140/90 | 100 | D | 36 |
| 9 | 44 | F | 4 | 24 L | Vulval abscess | 36 | 150/85 | 130 | N | $38^{5}$ |
| 10 | 39 | M | 1 | 36 L | Incorrect insulin | 24 | 120/80 | 95 | N | $36^{5}$ |
| 11 | 19 | F | — | — | UTI | 30 | 100/65 | 115 | D | $38^{5}$ |
| 12 | 26 | F | 6 | 30 MIX | Decreased insulin | 36 | 105/70 | 105 | N | 37 |
| 13 | 20 | M | — | — | None found | 24 | 80/? | 125 | N | 36 |
| 14 | 36 | M | 2 | 74 MIX | Myocardial infarct. Chest infection | 40 | 150/95 | 92 | C | 40 |
| | | | | | Hyperglycaemic non-ketotic | | | | | |
| 15 | 16 | F | — | — | Cellulitis | 16 | 105/65 | 82 | N | 38 |
| 16 | 30 | M | 2 | 88 MIX | 'Flu' | 18 | 135/85 | 90 | N | $38^{5}$ |
| | | | | | Hyperosmolar non-ketotic | | | | | |
| 17 | 68 | F | 10 | Diet | CVA | 30 | 170/105 | 104 | C | $36^{5}$ |
| 18 | 84 | M | 24 | Diet | Pneumonia | 36 | 160/100 | 88 | PC | 38 |
| 19 | 73 | F | 12 | Chlor. | F. femur CVA | 20 | 80/? | 120 | C | $38^{5}$ |
| 20 | 80 | F | 8 | Chlor. | F. femur DVT | 24 | 100/60 | 106 | D | $36^{5}$ |
| 21 | 79 | F | 13 | Chlor. | Pneumonia | 35 | 130/85 | 113 | C | 38 |
| 22 | 65 | M | 12 | 46 MIX | None found | 28 | 110/80 | 92 | N | 37 |

**Key**

L — Lente insulin

MIX — soluble and isophane insulin mixture

UTI — Urinary tract infection

DVT — Deep venous thrombosis

F — Fractured

Chlor. — Chlorpropamide 250 mg daily

CVA — Cerebro-vascular accident

RR — Respiratory rate per minute.

BP — Blood pressure mm Hg

N — Normal

D — Drowsy

PC — Pre-coma

C — Coma

DM — Diabetes Mellitus

### TABLE 2

### BIOCHEMICAL STUDIES ON ADMISSION

| Patient | Plasma glucose mmol/l | Plasma sodium mmol/l | Plasma potassium mmol/l | Plasma bicarbonate mmol/l | pH | Ketostix | Plasma urea mmol/l | Osmolarity mosm/l |
|---|---|---|---|---|---|---|---|---|
| 1 | 34.1 | 144 | 4.8 | 12 | 7.31 | +++ | 16.7 | 348 |
| 2 | 44.5 | 136 | 4.0 | 9 | 7.25 | +++ | 7.0 | 332 |
| 3 | 26.3 | 133 | 3.8 | 6 | 7.12 | +++ | 11.3 | 311 |
| 4 | 11.1 | 129 | 2.6 | 5 | 6.95 | +++ | 8.3 | 283 |
| 5 | 52.3 | 136 | 3.9 | 16 | 7.31 | +++ | 14.5 | 347 |
| 6 | 59.0 | 131 | 2.7 | 8 | 7.01 | +++ | 8.3 | 332 |
| 7 | 36.8 | 128 | 2.4 | 7 | 7.05 | +++ | 8.0 | 306 |
| 8 | 60.0 | 132 | 4.7 | 19 | 7.37 | ++ | 8.3 | 342 |
| 9 | 42.1 | 142 | 3.9 | 11 | 7.28 | ++ | 12.2 | 346 |
| 10 | 31.5 | 137 | 4.8 | 10 | 7.02 | +++ | 18.7 | 334 |
| 11 | 21.0 | 138 | 4.0 | 9 | 7.12 | +++ | 7.7 | 314 |
| 12 | 18.7 | 142 | 4.9 | 7 | 7.09 | +++ | 10.7 | 323 |
| 13 | 42.2 | 137 | 3.1 | 9 | 7.11 | +++ | 6.0 | 328 |
| 14 | 24.2 | 135 | 2.9 | 6 | 6.97 | +++ | 12.0 | 312 |
| 15 | 44.7 | 132 | 4.9 | 22 | 7.28 | 0 | 10.0 | 329 |
| 16 | 36.5 | 130 | 5.0 | 27 | 7.35 | + | 8.0 | 312 |
| 17 | 36.8 | 148 | 5.7 | 19 | 7.21 | 0 | 14.5 | 359 |
| 18 | 81.0 | 142 | 5.1 | 18 | 7.37 | 0 | 16.7 | 387 |
| 19 | 49.7 | 158 | 4.7 | 16 | 7.24 | + | 22.5 | 393 |
| 20 | 31.3 | 144 | 5.8 | 22 | 7.37 | 0 | 20.8 | 352 |
| 21 | 37.4 | 139 | 4.9 | 16 | 7.29 | 0 | 25.0 | 350 |
| 22 | 44.0 | 139 | 5.4 | 9 | 7.21 | 0 | 11.0 | 344 |

**References**

Alberti, K. G. M. M. and Hockaday, T. D. R. (1972) Rapid blood ketone body estimation in the diagnosis of diabetic ketoacidosis. British Medical Journal, **2**, 565.

Alberti, K. G. M. M., Hockaday, T. D. R. and Turner, R.C.(1973). Small doses of intramuscular insulin in the treatment of diabetic "coma". Lancet, **2**, 515.

Kidson, W., Casey, J., Kraegen, E. and Lazarus, L. (1974). Treatment of severe diabetes mellitus by insulin infusion. British Medical Journal, **2**, 691.

Page, M. McB., Alberti, K. G. M. M., Greenwood, R., Gumaa, K. A., Hockaday, T. D. R., Lowy, C. Nabarro, J. D. N. Pyke, D. A. Sönksen, P. H. Watkins, P. J. and West, T. E. T. Treatment of diabetic coma with continuous low-dose infusion of insulin. British Medical Journal, **2**, 687.

Semple, P. F., White, C. and Manderson, W. G. (1974). Continuous intravenous infusion of small doses of insulin in treatment of diabetic keto-acidosis. British Medical Journal, **2**, 694.

## Figures and Tables

**Figure 1. f1:**
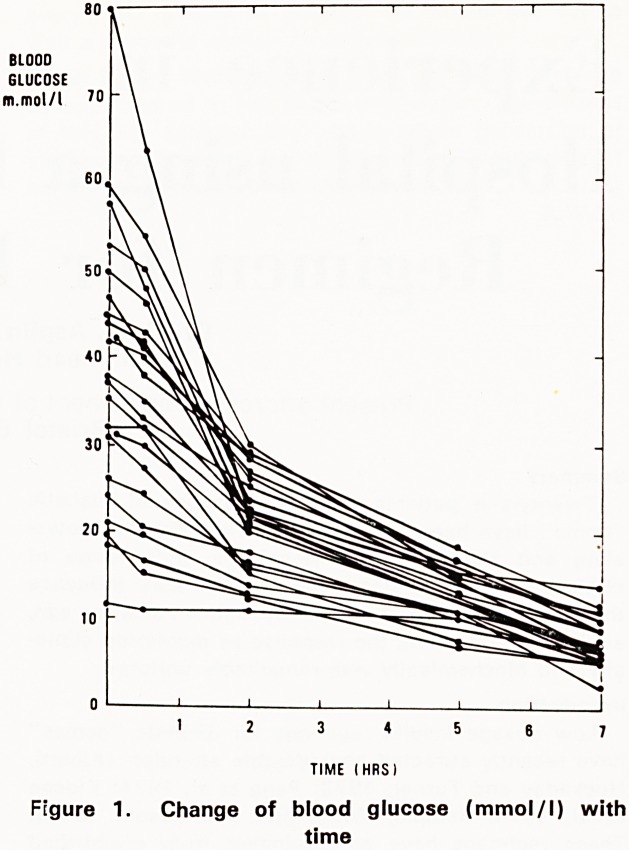


**Figure 2. f2:**